# Spatio-Temporal Evolution and Obstacle Factors Analysis of Tourism Ecological Security in Huanggang Dabieshan UNESCO Global Geopark

**DOI:** 10.3390/ijerph19148670

**Published:** 2022-07-16

**Authors:** Mengting Chen, Liang Zheng, Dike Zhang, Jiangfeng Li

**Affiliations:** 1 School of Public Administration, China University of Geosciences, Wuhan 430074, China; chenmt@cug.edu.cn; 2 Changjiang Institute of Survey, Planning, Design and Research, Wuhan 430014, China; zl@cug.edu.cn; 3 Key Laboratory of Changjiang Regulation and Protection of Ministry of Water Resources, Wuhan 430014, China; 4 School of Foreign Languages, China University of Geosciences, Wuhan 430074, China

**Keywords:** tourism ecological security, Driving-Pressure-State-Impact-Response (DPSIR) model, spatial autocorrelation, obstacle analysis, Huanggang Dabieshan UNESCO Global Geopark (UGGp)

## Abstract

The United Nations Educational, Scientific and Cultural Organization (UNESCO) Global Geoparks (UGGp) and geotourism activities not only improve people’s scientific quality by popularizing geoscience knowledge, but also play important roles in protecting precious geoheritages and promoting the development of regional economies. However, tourism activities also have a negative impact on the local ecological environment, placing the regional ecological system under great pressure. Therefore, this paper constructed a tourism ecological security evaluation indicator system suitable for geoparks by using the “Driving-Pressure-State-Impact-Response” (DPSIR) model. The spatial autocorrelation and obstacle degree model are used to analyze the spatio-temporal characteristics and influencing factors of the tourism ecological security index (TESI) of Huanggang Dabieshan UGGp in 2000, 2005, 2010, 2015 and 2018, respectively. The results indicate that the TESI of the study area has gradually improved from 2000 to 2018. Spatially, the level of TESI presents a gradient distribution from the townships where the main scenic spots are located to the surrounding townships. The main obstacle factors affecting TESI include: per capita tourism income, proportion of comprehensive tourism revenue in GDP, per capita net income of rural residents, proportion of tertiary industry in GDP, coverage of nature reserves, planning integrity of geopark, informatization of geopark, growth rate of tourists, comprehensive utilization rate of solid waste, etc. The influencing factors of TESI varied from time to time. Balancing the conflict between local tourism activities and environmental protection, encouraging the participation of local communities, and strengthening science popularization for the local public will effectively improve the tourism ecological security of geoparks.

## 1. Introduction

In recent decades, the vigorous development of the tourism industry has brought a lot of environmental pressure and influence, which seriously threaten ecological security and has received great attention. The development of tourism destinations, tourism resources and tourism markets has promoted the rapid development of the regional economy, increased foreign exchange income; promoted the development of the service industry, provided a large number of jobs, improved infrastructure, and increased the visibility of tourism destinations [1]. However, tourism has brought enormous pressures and risks on the local ecological environment mainly caused by the development of tourism resources, the construction of scenic spots, the passenger flow of tourists, and so on [1,2].

According to statistics, tourism has contributed much to global greenhouse gas emissions, accounting for 12.5% of total global emissions [3]. Research shows that emissions caused by tourism are projected to double from 2005 to 2035 [4]. Tourism activities such as transportation and accommodation involve higher energy consumption and carbon intensity, which have a big impact on the climate [5,6]. Moreover, some tourism activities have directly interfered with the local flora and fauna communities and the associated ecosystems, and disturbed the habitats of birds [7,8,9]. The consumption of resources and the destruction of ecological environments not only affect the diversity and functionality of the ecosystems of tourism destinations, but also seriously threaten the ecological security of these places.

Ecological security is an emerging research field in recent years, which has received extensive attention from academics [10,11,12,13,14]. It refers to the state in which an ecosystem provides material resources and services for the survival of human society and promotes economic development on the basis of ensuring its own integrity and health [15]. Ecological security assessment research has been carried out from national and regional perspectives [16,17,18,19,20]. The research objects include cities, land, river basins, ecologically fragile areas, ecological protection areas and so on [21,22,23,24,25,26,27,28]. Tourism ecological security is the concrete practice of ecological security in the tourism discipline. The concept of ecological security has been integrated into research on ecotourism and sustainable tourism for a long time [29,30,31,32]. Many studies have shown that tourism development can balance conflicts between socio-economic development and environmental protection in these regions to a certain extent [33]. Therefore, the ecological security of tourism destinations has always been a subject of widespread concern [34,35,36]. At the same time, it is also one of the important fields in the study of tourism destination sustainable development [35]. Consequently, tourism ecological security can be roughly summarized as a state that, through the rational development of tourism resources and the governance of ecological environment, the ecosystem of tourism destination keeps structural stability and functional diversity, provides a rich material foundation and environmental space for tourism development, and maintains the coordinated and sustainable development of the nature-society-economy complex ecosystem.

The study of tourism ecological security involves the fields of ecology, tourism science, geography, environmental science, energy science, etc. [37,38,39,40,41]. Early studies on tourism ecological security mainly focused on the impact of tourism activities on the environment, including tourism environmental capacity, tourism environmental protection, tourism carrying capacity and sustainable tourism [30,42,43,44,45,46,47]. Subsequently, more research has been done on the evaluation and measurement, spatial and temporal pattern, driving mechanism, prediction and early warning of tourism ecological security [48,49,50,51,52,53].

The evaluation of tourism ecological security mainly focuses on evaluation indicators and methods. The “Pressure-State-Response” (PSR) model, “Driving-Pressure-State-Impact-Response” (DPSIR) model, “Pressure-State-Response-Environment-Economy-Society” (PSR-EES) model, and other quantitative models are usually used to establish the evaluation indicator system of tourism ecological security [25,49,54,55]. Quantitative research methods mainly include tourism environmental carrying capacity, ecological footprint (EF) method, comprehensive index method, analytic hierarchy process (AHP) method, improved Technique for Order Preference by Similarity to Ideal Solution (TOPSIS) method, grey relation projection method, and so on [27,56,57,58,59,60]. The grey correlation degree and obstacle degree model are usually used to analyze influencing factors [53,61,62]. Compared with other evaluation models, the DPSIR model is more comprehensive, logical and systematic. It has high applicability for tourism ecological security evaluation [50], which can effectively identify the operating status of the tourism ecosystem. It can not only fully reflect the interactive relationship among human activities, the ecological environment and socioeconomic development, but also indicate the cyclic characteristics of system development [51]. The obstacle degree model can quantify the obstruction degree of influencing factors [63], which is conducive to accurately identifying the main obstacle indicators for further evaluation. Therefore, they are selected as the research methods for this paper.

From the existing literature, the evaluation and methods of tourism ecological security usually depend on the research object. Therefore, it is necessary to establish an appropriate evaluation indicator system [27]. Many kinds of tourism destinations have been studied, e.g., forest, lake, mountain areas, island and other different types of tourism destinations [48,58,64,65,66]. However, little research has been focused on geoparks, which aim to achieve geoheritage conservation and regional sustainable development. The United Nations World Tourism Organization (UNWTO) recommended five central pillars for sustainable development through tourism in 2017, including inclusive and sustainable economic growth, employment and poverty alleviation, environmental protection and climate change, heritage and cultural values, mutual understanding, peace, and security [47]. The United Nations Educational, Scientific and Cultural Organization (UNESCO) proposed the establishment of the Global Geoparks Network (GGN) in 1999 to manage and protect geoheritages and landscapes of international geological significance, and advocate the sustainable utilization of natural resources and sustainable tourism [67]. The “International Geoscience and Geoparks Programme (IGGP)” was officially approved in 2015, updating GGN to UNESCO Global Geoparks (UGGp). The Geoparks Initiative highlights the potential for interaction between the development of social economy and culture and the conservation of eco-environment [68]. At present, the research on geoparks is mainly focused on the classification and evaluation of geoheritage resources, geoheritage characteristics and geomorphological formation processes, tourist behavior characteristics and perception, geotourism projects design and geoproducts innovation, local community participation, etc. [69,70,71,72,73,74,75]. In fact, with the development of geopark construction and the increase in tourism activities, the local ecosystem faces great pressure. Thus, this paper aims to establish a tourism ecological security evaluation indicator system for geoparks, to enrich the theory of tourism ecology and ecological security. It can provide feasible paths and improvement measures for the sustainable development of geoparks through the tourism ecological security evaluation.

In this paper, Huanggang Dabieshan UGGp is selected as a typical case for the following reasons. Firstly, the widely distributed geoheritages and landscapes in Huanggang Dabieshan UGGp are of great international significance in terms of geological and ecological aesthetics. The study area is an important part of the “geological-geographical-climatic-ecological” dividing line in eastern China. It is also rich in biodiversity, which is a relatively well-preserved storehouse of species resources in Central China. Secondly, in the past decade, with the rapid development of tourism, both the landscape and the ecosystem of Huanggang Dabieshan UGGp have been under pressure from the consumption of tourism resources and human activities. It has posed a great threat to the geoheritages and ecosystems. Thirdly, compared with other tourism destinations, geoparks have their unique features, mainly in the unique geotourism resources and geological science popularization and education functions. Finally, few studies on tourism ecological security take geoparks as the object so that this paper tries to fill the gap and puts forward some suggestions for reference.

Therefore, the purpose and significance of this paper are as follows: (1) establish a tourism ecological security evaluation model for Huanggang Dabieshan UGGp. A comprehensive multi-factors evaluation indicator system based on the DPSIR model is constructed and some evaluation indicators are selected that are different from other tourism destinations, which can enrich the theoretical research of geopark and ecological security. (2) explore the tourism ecological security index (TESI) of Huanggang Dabieshan UGGp and its spatio-temporal distribution characteristics. The spatial and temporal distribution trend is obtained through spatial autocorrelation analysis. The changes of the TESI level throughout the study area are analyzed in different dimensions. (3) diagnose the main influencing factors affecting tourism ecological security. The core factors affecting the TESI of Huanggang Dabieshan UGGp in different periods are identified through an obstacle degree model. (4) discuss the countermeasures and suggestions for ecotourism and sustainable development of geoparks, so as to provide theoretical guidance for Huanggang Dabieshan UGGp and other geoparks to coordinate the relationship between conservation and tourism development. It is conducive to promote the formulation and implementation of relevant policies.

**Figure 1:**
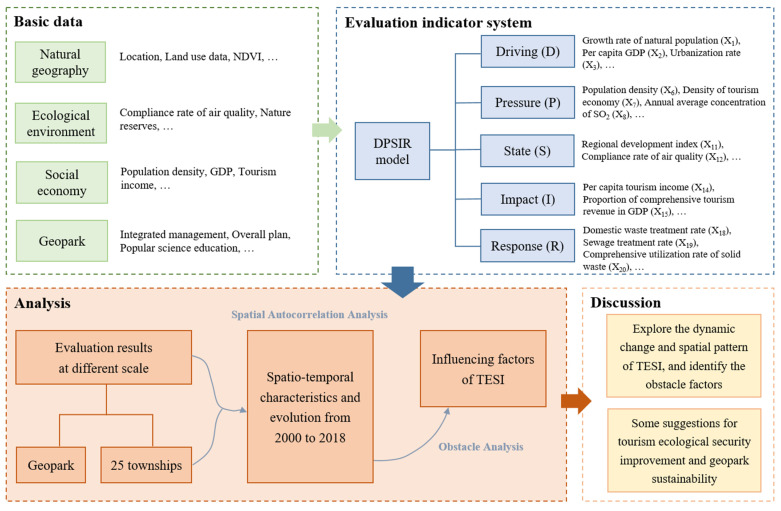
The framework of tourism ecological security evaluation of Huanggang Dabieshan UGGp.

The research framework is shown in Figure 1.

## 2. Materials and Methods

### 2.1. Study Area

**Figure 2:**
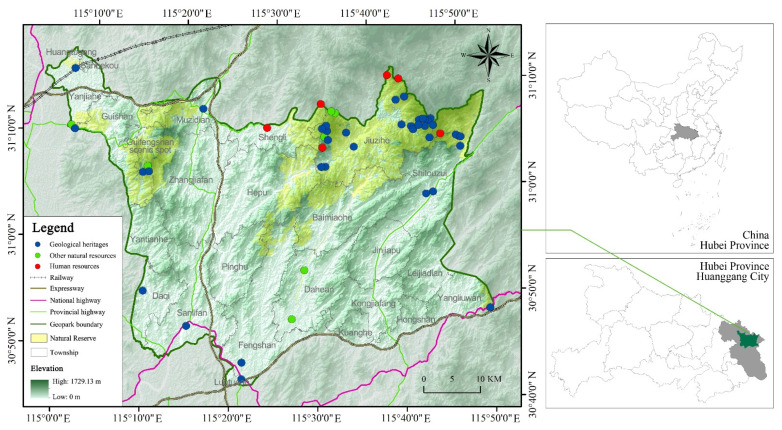
Geographical location of Huanggang Dabieshan UGGp.

Huanggang Dabieshan UGGp is situated in Eastern Asia, Huanggang City, Hubei Province of the People’s Republic of China, with a total area of 2625.54 square kilometers (Figure 2). The administrative division of the study area involves Macheng City, Luotian County and Yingshan County, including 25 townships.

Huanggang Dabieshan UGGp is characterized by a continental orogenic belt, a tectonic deformation metamorphic belt and granite mountain landforms. The terrain of the geopark gradually tilts from north to south. Among them, there are 96 peaks with an altitude of more than 1000 m in the north. The highest peak, at 1729.13 m above sea level, is located at the junction of Luotian County and Yingshan County in the northeast.

During the ongoing geological evolution, various typical geological landscapes have been formed in this area, which mainly includes 4 global-level, 5 national-level, 21 provincial-level and 23 local-level geoheritages. The unique location belonging to the subtropical monsoon climate zone produces excellent natural conditions, which have created a region with dazzling biodiversity. Huanggang Dabieshan UGGp boasts valuable geoheritages, unique ecological landscapes and beautiful cultural sights, which has made it a rare and significant geopark and geoheritages reserve in the world.

The study area was approved as a national geopark in 2009 and became a member of UGGps in 2018. As the main tourism destination in the east of Hubei Province and Huanggang City, Huanggang Dabieshan UGGp has attracted a large number of tourists from local and surrounding cities, and promoted the development of the local tourism economy and related service industries. The tourism economy in Huanggang City has grown at an annual rate of more than 20%, with Huanggang Dabieshan UGGp as the leading tourism industry [76]. In 2018, the number of tourists in Huanggang City increased to 36.45 million and total tourism revenue reached 3.85 billion dollars.

The main tourist areas of Huanggang Dabieshan UGGp include Tiantangzhai, Bodaofeng, Wujiashan, Guifengshan, Jiulongshan, etc. The typical geological tourist attractions are granite pictographic stone landscapes, including Philosopher Peak, Guifeng Peak (Stone Tortoise), Longtan Gorge, etc. Every spring and summer, Huanggang Dabieshan UGGp is crowded with tourists enjoying flowering rhododendrons and their summer vacation. According to incomplete statistics, the geopark’s ticket revenue alone reached 101 million dollars in 2018. The comprehensive tourism revenue reached 957million dollars, accounting for 31% of the regional Gross Domestic Product (GDP).

After more than a decade of tourism development, the huge tourist flow and the development of resources and tourism projects inevitably brought a series of negative impacts on the ecological environment. The evaluation of tourism ecological security in Huanggang Dabieshan UGGp can not only obtain the influencing factors affecting the environment in this area, but also explore the appropriate development direction of the geopark, and provide a scientific basis for the sustainable and healthy development of Huanggang Dabieshan UGGp, which has important research value and practical significance.

### 2.2. Data Sources

The land use data, spatial distributions of population density, GDP, Normalized Difference Vegetation Index (NDVI) and other data of the study area were obtained from the Resource and Environment Science and Data Center of Chinese Academy of Sciences (http://www.resdc.cn/, accessed on 5 April 2022). The administrative boundaries, nature reserves, and tourism resources came from field investigation and project statistics provided by Huanggang Dabieshan National Geopark Administrative Office.

Other socio-economic data were collected from *Hubei Statistical Yearbook*, *Huanggang Statistical Yearbook*, *China County Statistical Yearbook*, the environmental quality report of Huanggang City, etc. Some missing data were filled by the moving average method.

Due to the large sample size, this paper only selected five periods of data in 2000, 2005, 2010, 2015 and 2018 for analysis. The spatial and temporal distribution characteristics of TESI in the study area will be presented in 25 townships.

### 2.3. Methods

#### 2.3.1. Evaluation Indicator System for Tourism Ecological Security

Academics have established many indicator systems for evaluating research [25,55,60]. Among them, the “Driving-Pressure-State-Impact-Response” (DPSIR) model was established by the European Environment Agency (EEA) in 1993. It has integrated the “Pressure-State-Response” (PSR) model and the “Driving Force-State-Response” (DSR) model, and added “Impact” indicators in its framework [77]. The DPSIR model can effectively reflect the interaction between elements in a system. It has been widely applied to quantitative research, such as environmental assessment, water resources ecological security assessment, sustainable development capacity assessment, etc. [78,79,80].

**Figure 3:**
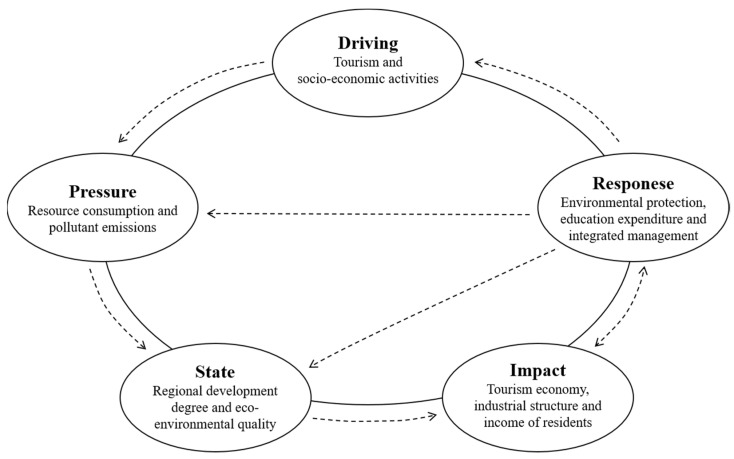
The operational mechanism of the DPSIR model.

In the tourism ecosystem, the DPSIR model can effectively measure the relationship between tourism activities and the ecological environment, and reflect the positive feedback of human society [51]. The operational mechanism of the DPSIR model can be summarized as follows [81,82,83,84]: as the long-term driving force (D) affecting the tourism ecological security, social and economic factors have imperceptibly caused various pressures (P) on the natural environment, ecology and social resources. These pressures (P) are directly reflected in the changes of the regional social economy and environmental state (S). Furthermore, it has a series of impacts (I) on the regional ecosystem, prompting human to take a series of positive response (R) measures to achieve the goal of sustainable development. At the same time, these response (R) measures not only act on the system composed of human economy and society (D), but also directly have a positive impact (I) on pressure (P) and state (S), so as to form a circular, closed loop (Figure 3).

Considering the impact of natural conditions, human activities, economic growth, social development and other factors on the tourism ecosystem of geopark, the DPSIR model is chosen as the evaluation model of the tourism ecological security of Huanggang Dabieshan UGGp. Combined with the previous literature, field investigation and interviews and acquired data [25,27,49,50,51,52,53,85], 25 indicators are selected.

Driving indicators represent human socio-economic activities. The growth of population, urbanization, national economy, and tourism demand are the most basic driving forces.

Pressure indicators describe the load of the exploitation on the ecological environment. The reasons for the change are given from the perspectives of population density, tourism economic density, and annual average concentration of pollutants.

State indicators reflect the ecological environment and socio-economic development of the study area. The regional development index is the ratio of the total area of regional cultivated land and construction land to the total area of regional land, reflecting the development state of human activities and urbanization process in a period. The compliance rate of air quality and NDVI indicate air quality status and vegetation coverage status, respectively.

Impact indicators refer to the indicators that bring changes to the maintenance of tourism ecological security and industrial development when the ecological environment or socio-economic system changes. Tourism revenue, rural residents’ income, and proportion of tertiary industry in GDP reflect that the higher the scale of tourism and tertiary industry is, the more investment will be made in improving tourism ecological security, and the greater the positive impact will be produced on TESI.

Response indicators reflect the positive measures taken by the government and the geopark to improve the regional tourism ecological security. Domestic waste treatment rate, sewage treatment rate, and comprehensive utilization rate of solid waste represent the situation of cleaner production, environmental treatment and the degree of resources recycling and reuse. The coverage of nature reserves reflects the degree of local government’s protection and attention to the ecological environment. The proportion of education expenditure in GDP indicates the importance of education development in the region, which indirectly reflects the education level of the local residents. Planning integrity, interpretive coverage and informatization of geopark refer to the management response of Huanggang Dabieshan UGGp. The better the management, the greater the role of promotion for the TESI. Since the study area is a geopark, the selection of indicators should be distinguished from other tourism destinations. The *UNESCO Global Geopark Applicant’s Evaluation Document A—Self Evaluation* is an assessment document officially released by UNESCO, which is referential [86]. The contents relate to overall planning, a science popularization interpretation system, and informatization construction are extracted and summarized into the last three indicators. The value is assigned by calculating the ratio of the total self-assessment score of planning, interpretation and informatization to the total standard score. As an official document that UGGps need to be evaluated every four years, it is applicable to all the geoparks.

The complete evaluation indicator system is shown in Table 1.

#### 2.3.2. Comprehensive Index Method

Because of the difference in dimension and order of magnitude, the original data need to be standardized. For the positive indicator,

(1) $$x_{ij}^{\prime }=\left(x_{ij}-x_{jmin}\right)/\left(x_{jmax}-x_{jmin}\right)$$

and for the negative indicator,

(2) $$x_{ij}^{\prime }=\left(x_{jmax}-x_{ij}\right)/\left(x_{jmax}-x_{jmin}\right)$$

In the above formulas, $x_{ij}^{\prime }$ stands for the standardized value of the original data; $x_{ij}$ stands for the original value of indicator *j* in year *i*; $x_{imax}$ and $x_{jmin}$ stands for the maximum and minimum values of indicator *j* among all years, respectively.

The entropy weight method is used to calculate the weight of each indicator in the evaluation system. It can analyze the degree of correlation between indicators based on objective information, and reduce the impact of subjective factors to a certain extent [19,53,58]. The formulas are as follows:

(3) $$p_{ij}=x_{ij}^{\prime }/\overset{n}{\underset{j=1}{\sum }}x_{ij}^{\prime }+0.000001$$

(4) $$E_{j}=-k\overset{n}{\underset{j=1}{\sum }}p_{ij}\ln p_{ij}$$

(5) $$w_{j}=\left(1-E_{j}\right)/\overset{n}{\underset{j=1}{\sum }}\left(1-E_{j}\right)$$

In the above formulas, $p_{ij}$ represents the proportion of the standardized value of indicator *j* in year *i* to the sum of all the standardized values of indicator *j*. Since $\ln p_{ij}$ is meaningless when $p_{ij}$ = 0, the formula is revised to (3).

$E_{j}$ represents the entropy of indicator *j*; *k* represents the Boltzmann constant, *k =* 1/ln(*n*); $w_{j}$ represents the information entropy weight of indicator *j*. The weights of all indicators are shown in Table 1.

The TESI can be calculated by the comprehensive index method. The formula is as follows:

(6) $$TESI_{i}=\overset{n}{\underset{j=1}{\sum }}w_{j}x_{ij}^{\prime }$$

where $TESI_{i}$ is the TESI in year *i*; $w_{j}$ and $x_{ij}^{\prime }$ are the weight and standardized value of indicator *j*; and *n* is the number of indicators in the evaluation system. The TESI level can be classified into 5 types [35,53,58]: unsafe, less unsafe, critical safe, relatively safe, and safe (Table 2).

#### 2.3.3. Spatial Autocorrelation Analysis

Spatial autocorrelation analysis is a method of exploratory spatial data analysis, which reveals the similarity and spatial correlation of attribute values of adjacent regions [50,51,87,88,89]. This method is usually measured by Moran’s *I*, including the Global Moran Index and the Local Moran Index. The interval of Moran’s *I* value ranges from −1 to 1. If Moran’s *I* value is greater than 0 and passes the autocorrelation significance test, it illustrates that the change trend of a spatial unit is the same as that of adjacent units. That is, the spatial autocorrelation is positive and there is aggregation. If Moran’s *I* value is less than 0, the spatial autocorrelation is negative. The larger the absolute value of Moran’s *I* is, the stronger the spatial autocorrelation will be. When the value is equal to 0, the spatial autocorrelation is random. The formulas are as follows:

(7) $$I_{G}=\overset{n}{\underset{i=1}{\sum }}\overset{n}{\underset{j\neq i}{\sum }}w_{j}\left(y_{i}-\bar{y}\right)\left(y_{j}-\bar{y}\right)/S^{2}\overset{n}{\underset{i=1}{\sum }}\overset{n}{\underset{j\neq i}{\sum }}w_{ij}$$

(8) $$I_{L}=\frac{y_{i}-\bar{y}}{S^{2}}\overset{n}{\underset{j\neq i}{\sum }}w_{ij}\left(y_{i}-\bar{y}\right)$$

where $I_{G}$ is the Global Moran Index; $I_{L}$ is the Local Moran Index; *n* is the number of spatial units; $y_{i}$ and $y_{j}$ are the observed values of spatial units; $\bar{y}$ is the average of the observed values; $S^{2}$ is the variance of the observed values; $w_{ij}$ is a weight matrix based on the spatial adjacency relationship.

#### 2.3.4. Obstacle Analysis

Obstacle factors refer to the barriers that restrict and hinder the tourism ecological security of geoparks. It is helpful to improve the level of TESI by evaluating the barrier effect of each indicator and finding out the main obstacle factors [58,62,90]. The obstacle degree model consists of three indexes: deviation degree ($I_{ij}$), factor contribution degree ($w_{ij}$) and obstacle degree ($O_{ij}$). The formula is as follows:

(9) $$I_{ij}=1-x_{ij}^{\prime }$$

(10) $$O_{ij}=I_{ij}w_{ij}/\overset{n}{\underset{j=1}{\sum }}I_{ij}w_{ij}\times 100\%$$

where $I_{ij}$ indicates the gap degree between the indicator *j* and the target of tourism ecological security; $w_{ij}$ is expressed by the weight of each indicator, which represents the contribution degree of a single factor to the overall objective of tourism ecological security; $O_{ij}$ is the obstacle degree of indicator *j* on tourism ecological security in year *i*.

## 3. Results

### 3.1. Spatio-Temporal Characteristics of TESI

#### 3.1.1. TESI of Huanggang Dabieshan UGGp

It is shown from Table 3 that the TESI of Huanggang Dabieshan UGGp has gradually increased from 2000 to 2018, and that the security level has improved from less unsafe to relatively safe.

According to the overall change of TESI in the study area, it can be divided into three stages. In the unsafe stage (2000-2005), the TESI increased very slowly from 0.176 to 0.227, which were all in the status of unsafe. Various social, economic and environmental problems have serious constraints on the tourism ecological security. In the critical safe stage (2010-2015), the TESI continued to grow from 0.44 to 0.551. The tourism ecological security of Huanggang Dabieshan UGGp has reached the status of critical safe from unsafe. In the relatively safe stage (2018), the TESI rose to 0.657. The study area is basically in a relatively safe status.

#### 3.1.2. Evolution of TESI Level in Different Townships

**Figure 4:**
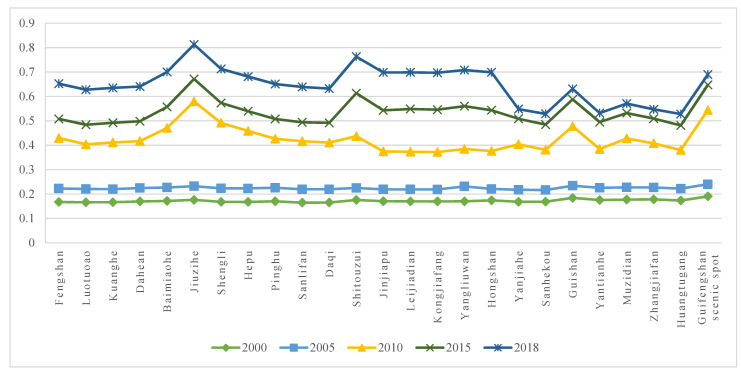
TESI of each township in Huanggang Dabieshan UGGp from 2000 to 2018.

**Figure 5:**
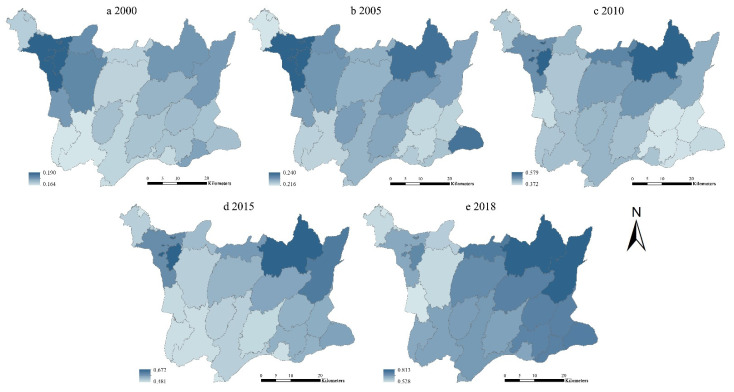
Distribution of TESI in Huanggang Dabieshan UGGp from 2000 to 2018.

Taking 25 townships as evaluation units, the TESI of each township is calculated and shown in Figure 4. It can be seen that, in terms of time, the TESI of each township has shown a steady upward trend. The TESI of all the townships was at a low level in 2000 and 2005, and then presented a multi-level development state since 2010. ArcGIS software is used for visualization, as shown in Figure 5.

### 3.2. Spatial Pattern Analysis

The Moran’s *I* value of TESI from 2000 to 2018 was calculated by GeoDa software. The Global Moran’s *I* values of these 5 phases were greater than 0, and passed the significance test of 5%. It revealed that the TESI in Huanggang Dabieshan UGGp from 2000 to 2018 had a significant positive correlation, which means it had obvious spatial distribution characteristics of aggregation. The townships with higher TESI tended to be adjacent, as did the townships with lower TESI.

From the perspective of time series, Moran’s *I* value showed a “W” trend of increasing fluctuation, and reached its highest level in 2018. The decrease in Moran’s *I* value showed that the uniformity of TESI distribution in Huanggang Dabieshan UGGp had reduced, as townships with changes in TESI had increased. The increase in Moran’s *I* value indicated that the level of TESI had been increased and the uniformity of TESI distribution had been improved. The spatial correlation of TESI had been gradually strengthened, as the distribution of TESI tended to be stable.

**Figure 6:**
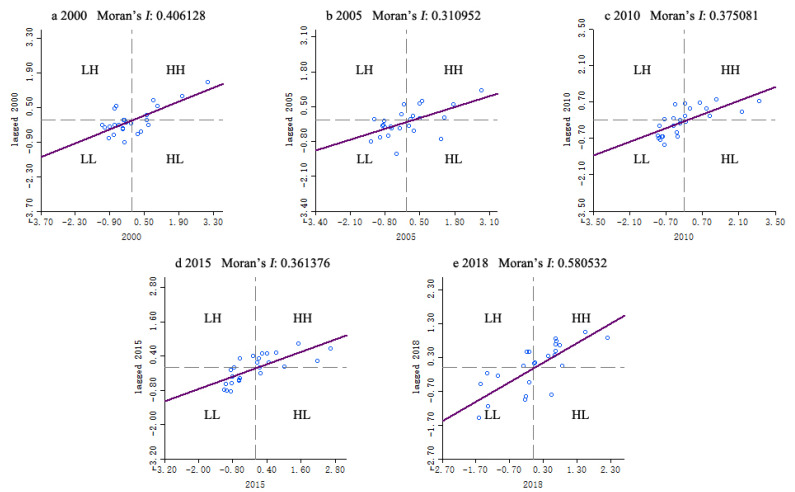
Moran scatter plot of TESI in Huanggang Dabieshan UGGp from 2000 to 2018. HH, High-High agglomeration; HL, High-Low agglomeration; LL, Low-Low agglomeration; LH, Low-High agglomeration.

As illustrated in Figure 6, the TESI showed an obvious spatial disparity. The TESI of most townships were in “High-High (HH)” and “Low-Low (LL)” quadrants, indicating that it had strong local autocorrelation and the overall pattern was relatively stable. Most townships were surrounded by townships with similar security level; that is, it had a strong spatial dependence.

**Figure 7:**
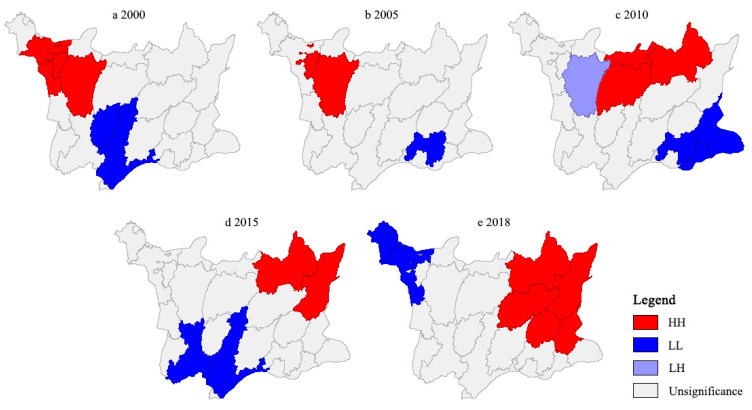
Local indicators of spatial autocorrelation (LISA) cluster maps of TESI in Huanggang Dabieshan UGGp from 2000 to 2018. HH, High-High agglomeration; LL, Low-Low agglomeration; LH, Low-High agglomeration.

According to Figure 7, the TESI generally presented dynamic spatial agglomeration with “HH” type and “LL” type, which showed positive local spatial autocorrelation. From 2000 to 2018, “HH” agglomeration was successively concentrated in the Guifengshan scenic spot, Guishan, Shengli, Jiuzihe, and Shitouzui, which were little different to the neighboring townships and belonged to local homogeneous distribution. “LL” agglomeration was successively distributed in Fengshan, Kongjiafang, Leijiadian, Sanlifan, and Yanjiahe. “Low-High (LH)” agglomeration indicated the low level of TESI in “LH” township and high level of TESI in neighboring townships, which only existed in Zhangjiafan in 2010. “High-Low (HL)” agglomeration showed the high level of TESI in “HL” township and low level of TESI in neighboring townships, presenting an obvious polarization effect and negative correlation. It did not exist in the study area throughout the study period.

### 3.3. Obstacle Factors of TESI

This paper only lists the top 8 obstacle factors of obstruction degree each year due to lack of space. As shown in Table 4, the main obstacles to the tourism ecological security of Huanggang Dabieshan UGGp included: per capita tourism income (X_14_), proportion of comprehensive tourism revenue in GDP (X_15_), per capita net income of rural residents (X_16_), proportion of tertiary industry in GDP (X_17_), coverage of nature reserves (X_21_), planning integrity of geopark (X_23_), informatization of geopark (X_25_), growth rate of tourists (X_4_), comprehensive utilization rate of solid waste (X_20_), etc.

Among these influencing factors, per capita tourism income (X_14_), proportion of comprehensive tourism revenue in GDP (X_15_), and per capita net income of rural residents (X_16_) were the main obstacles to the tourism ecological security of the geopark from 2000 to 2015. The impact of per capita tourism income (X_14_) was strong and remained a major barrier until 2015.

Coverage of nature reserves (X_21_), planning integrity of geopark (X_23_) and informatization of geopark (X_25_) were the main obstacles to tourism ecological security from 2000 to 2010.

From 2000 to 2005, domestic waste treatment rate (X_18_) and interpretive coverage of geopark (X_24_) showed that poor ecological environment quality and weak management also had a certain impact on tourism ecological security.

The common obstacles in 2010-2015 were: growth rate of tourists (X_4_), proportion of tertiary industry in GDP (X_17_), and comprehensive utilization rate of solid waste (X_20_). The common barriers in 2015-2018 were natural population growth rate (X_1_) and annual average concentration of SO_2_ (X_8_).

The greatest barriers in 2015 and 2018 were per capita tourism income (X_14_) and comprehensive utilization rate of solid waste (X_20_), respectively. In 2018, per capita GDP (X_2_), density of tourism economy (X_7_), regional development index (X_11_), compliance rate of air quality (X_12_), comprehensive utilization rate of solid waste (X_20_) and proportion of education expenditure in GDP (X_22_) also became important obstacle factors to tourism ecological security.

## 4. Discussion

### 4.1. Selection of Evaluation Indicators

In order to explore the reasons and trends of tourism ecological security, the material basis and ecological environmental conditions, which were provided by the tourism destination ecosystem for tourism and socio-economic development, should be comprehensively evaluated. A multi-dimensional evaluation is carried out by taking into account the pressure of resource consumption and emission brought about by tourism development and human social activities, as well as the response and maintenance measures taken by socio-economic systems and geoparks. By summarizing the existing literature and field investigations, and considering the availability of data, the indicators selected in this paper included the development of geopark tourism, population, economy, current situation of ecological environment, environmental protection and governance, etc. Most of the indicators had a high occurrence rate in the existing literature.

The driving indicators were selected from the aspects of geopark tourism and socio-economic development [84]. The growth rate of tourists and the growth rate of comprehensive tourism revenue represented the development of the tourism industry, reflecting that the more popular and attractive the geopark is to tourists, the higher the indirect threats to the ecosystem would be. The growth rate of natural population, per capita GDP and urbanization rate indicate the regional social and economic development, which can indirectly reflect the negative impacts of socio-economic development on regional resources consumption and ecological environment.

The pressure indicators are selected from the damage caused by tourism and human activities to the ecosystem. Population density indicates that resources consumption will increase ecological and environmental problems. Some scholars take tourism economic density as an impact indicator [50,51], but this paper holds that the density of tourism economy is the tourism economic income carried on the unit land, which indirectly reflects the consumption of regional resources and the threat faced by ecological environment, so it is used as a pressure indicator. The emission of pollutants also shows the negative damage of economic development on regional ecological environment. Similar indicators such as industrial wastewater discharge, industrial SO_2_ emission, industrial smoke dust emission and total exhaust emission should be selected based on the availability of data [22,49,50,52].

The state indicators were selected from the urbanization process and the health degree of ecological environment. The regional development index reflects human activities and the process of urbanization. The existing literature also used indicators such as the number of star hotels and the number of tourism practitioners [27,49]. This study did not conduct a complete survey of all the townships in the study area, so the data cannot be supported. Future research can consider these indicators that reflect the current situation of tourism development. The compliance rate of air quality and NDVI reflect the degree of air pollution and vegetation coverage in the region. Indicators such as per capita green area and green coverage rate of built-up areas are mostly used for urban and other research areas [27,50,52]. The study area in this paper is located in mountainous areas, with good overall vegetation coverage and relatively less construction land, so only NDVI was used. Forest coverage index, biodiversity index and ecological vulnerability index can be used to reflect the status of ecological resources in the future work.

The impact indicators reflect the impacts and changes of natural ecology and social resources under pressure, which are usually expressed in terms of per capita tourism income, per capita net income of rural residents, proportion of comprehensive tourism revenue in GDP and proportion of tertiary industry in GDP [50,51,52,53]. These indicators usually reflect positive impacts. Geoparks that benefit from tourism activities will pay more attention to the capital investment in tourism development and ecological environmental protection. Residents who benefit from tourism activities will cherish and participate in the tourism industry, and form a mutually beneficial and win-win situation with the geopark. The tertiary industry reflects the regional industrial structure. It is dominated by the service industry, with less resource consumption and light environmental pollution. The larger the proportion of the tertiary industry, the less damage and threat the ecological environment will suffer from.

The response indicators are selected from the positive measures taken by the government and managers to improve the regional tourism ecological security. The treatment of domestic waste, sewage and solid waste is the key factor for the sustainability of the ecological environment, so that the related indicators are used frequently. The coverage of nature reserves indicates the degree of local government’s attention to the ecological environmental protection. The number of college students has been used to reflect the education level of local residents [51,52]; this paper replaced it by the proportion of education expenditure in GDP due to the difficulty of data acquisition. The problems they reflect are basically the same, which mean the long-term measures of talent education. Some studies have used the proportion of investment in environmental protection [52,58], which is not used in this paper due to incomplete statistical data, but is necessary to be considered in the future. The indicators of management response of the geopark are selected according to the characteristics of the study area, so that other study areas should choose the corresponding indicators on the basis of their own actual situation.

In summary, the indicators of tourism ecological security evaluation should be scientific and reasonable, and have been widely accepted and used. Indicators that are relatively important, frequently used and proved useful in the existing literature should be adopted. The characteristics of the study area need to be reflected. The availability of data needs to be sufficiently considered.

### 4.2. Dynamic Change of TESI Level

Consistent with previous research, with the development of tourism economy, the TESI of Huanggang Dabieshan UGGp generally shows an upward trend [27,51,53]. When the geopark had not been established, the study area was dominated by traditional extensive agriculture. The awareness of ecological and environmental protection was weak, and the TESI level of the region was low. Since completion of the national geopark in 2009, in order to support the tourism development, build the tourism brand of Huanggang City and Eastern Hubei Province, the government of Huanggang City paid enough attention to the construction of the geopark and invested enough funds to comprehensively improve the ecological environment and ecotourism development. The tourism ecological security level of the study area has improved since 2010. In order to apply for UGGp and make tourism more ecological, the management agency formulated a reasonable development plan since 2013, such as the strengthening of management, the construction of a tourism talent team, the improvement of supporting infrastructure and the formulation of regulations on the protection of geoheritages and other resources.

At the same time, with the implementation of the 11th five-year plan, the construction of ecological civilization, the strategy of “two circles and one belt”, and the construction of “Ecological Hubei”, resource-saving and environment-friendly society became the main objective of current development [22], which contributed to the gradual improvement of the ecological environment in Huanggang City and the study area.

Moreover, with the popularization of basic education in China, the scientific and cultural quality of the resident population is constantly improving. It has been verified that a high level of educational attainment can promote pro-environmental behavior [91,92]. Accordingly, it has enhanced the tourism ecological awareness of the majority of local residents and tourists, and promoted the tourism ecological security of the study area to a certain extent.

From the development level of each township, the townships with a high level of TESI are the main scenic spots of Huanggang Dabieshan UGGp. With the development of geopark construction and tourism activities, more and more attention has been paid to these townships, and supporting measures such as investment, management and protection have also gradually been followed up. Furthermore, Huanggang Dabieshan UGGp has carried out a lot of work in the fields of geoscience popularization and education, ecological environmental protection, and township renovation in order to apply for UGGp. Therefore, the improvement of tourism ecological security is more obvious than in other townships. In addition, in townships with high security levels, there are various kinds of nature reserves, such as Dabieshan National Nature Reserve, Wujiashan National Forest Park, Zhangjiazui National Wetland Park, Tiantanghu National Wetland Park, etc. The natural conditions also have an important effect on the TESI of each township in Huanggang Dabieshan UGGp.

Townships with low TESI have also been positively affected by the development of the tourism economy and the comprehensive tourism income has increased continuously, but at the same time, it has also put pressure on tourist flow and the ecological environment. Due to the distribution of more cultivated land and construction land, higher population density, more resource consumption, and lower or no coverage of nature reserves, these townships have a relatively low level of TESI compared with other townships in the same period.

### 4.3. Spatial Pattern of TESI

Previous research has found that tourism ecological security has spatial dependence and spatial correlation, and the spatial spillover effect is obvious [50,93]. In this paper, the fluctuation of Moran’s *I* value of TESI indicates that the spatial correlation and agglomeration of tourism ecological security are increasing, which is consistent with the previous research results. The TESI level of a township is not independent in geographical space. It is often affected by neighboring townships and has a spatial interaction effect. Most townships are surrounded by townships with a similar security level, which also shows that tourism ecological security has spatial dependence and spillover effect.

“HH” agglomeration areas are mainly distributed in the Guifengshan scenic spot, Shengli, Jiuzihe, and Shitouzui. With the passage of time, the agglomeration area has transferred from northwest to northeast, which has played an active role in promoting the security level of surrounding townships, with radiation and spatial diffusion effect. The “LL” agglomeration area is mainly concentrated in Fengshan, Kongjiafang, Leijiadian, Sanlifan, and Yanjiahe. It has undergone several changes with the trend of “southwest-southeast-southwest-northwest”, which were jointly affected by macro policies and natural resource conditions. Only Zhangjiafan is “LH” type in 2010 because of the lack of outstanding tourism resources, excellent natural conditions and regional cooperation. Although it was adjacent to a township with a high level of TESI, it had not been driven by spatial dependence and spillover.

The great differences in the TESI of townships indicate that the regional tourism ecological security has obvious spatial differentiation characteristics, which accords with the conclusion of Ruan et al. [51]. In addition, in accordance with the previous research, economic advantages have an important impact on tourism ecological security [51,94]. There are more tourism activities in the townships where the tourist attractions are located. The development level of tourism economy here is higher, so there is more capital investment and maintenance. Therefore, the TESI level of these townships is relatively higher than that of other townships.

### 4.4. Identification of Obstacle Factors

It is profitable to penetrate into the restrictive factors and driving mechanisms of the tourism ecological security level in a region by obstacle analysis. A decision-making basis for tourism industry development, ecological environment protection, and industrial structure adjustment in geopark and surrounding areas can be provided in the future, too.

During the study period, tourism economic factors and ecological factors have played an important role in TESI, which is consistent with Tang’s conclusion [27]. Through the sorting and comparison, it can be seen that before the establishment of the geopark, the level of socio-economic development of the study area was relatively low, and the tourism industry had not yet started. The contribution of tourism economy was particularly small, accounting for only 1% of regional GDP. The overall planning of regional development was insufficient. Little attention was paid to ecological environmental protection. In addition, the low living standard of local residents led to their lack of awareness of environmental and ecological protection. A series of reasons had restricted the TESI of the research area during 2000 to 2005. Accordingly, per capita tourism income, proportion of comprehensive tourism revenue in GDP, per capita net income of rural residents, proportion of tertiary industry in GDP, domestic waste treatment rate, coverage of nature reserves, planning integrity of geopark, interpretive coverage of geopark, and informatization of geopark were important influencing factors.

With the construction of Huanggang Dabieshan UGGp in 2013 and the development of the tourism industry, the economic benefits brought by tourism were increasing gradually, and the investment indirectly used for development and protection was increasing. The growth of the proportion of tertiary industry in GDP reflected the optimization of industrial structures. Services and business-oriented industries consumed fewer resources and produced less environmental pollution, thus causing less damage and fewer threats to the ecological environment. All of these made the obstruction degree of indicators which are related to tourism and economy showed a downward trend in 2015. However, at the initial stage of geopark construction, the number of tourists showed an explosive growth, reflecting the lack of tourists’ density control, making the growth rate of tourists the most important obstacle factor in 2015.

With the rapid growth of tourism income, the resource consumption caused by the investment in tourism development and urbanization had been increasing, which had led to the aggravation of environmental pollution and the deterioration of ecological quality to a certain extent. These changes indicated that resource utilization and ecological environmental management were facing increasing pressure. This had been confirmed by the research of York, Tang and Wang et al. [2,95,96]. Therefore, density of tourism economy, annual average concentration of SO_2_, regional development index, compliance rate of air quality, and comprehensive utilization rate of solid waste were important factors hindering tourism ecological security in 2018. Overall, the obstruction degree of these socio-economic factors, although the main hindrances, were decreasing relative to the earlier period because TESI was generally developing in a good direction.

In addition, the proportion of education expenditure in GDP became a more significant barrier in 2018, suggesting that local education spending had remained at the same level for a long time. Although the general public was constantly becoming better educated, the disadvantage of the proportion of education expenditure in GDP was obvious when the economic and ecological indicators had noticeably improved. Consequently, the government needed to pay more attention to public education. The high scientific and cultural quality levels of residents will encourage their behavior to be more civilized, which will be more beneficial to the promotion of TESI of Huanggang Dabieshan UGGp.

### 4.5. Policy Implications

Over the past 20 years, China has made unremitting efforts to promote the construction of geoparks. By the end of 2021, 41 geoparks in China have become members of UGGps, and 281 geoparks have been officially named National Geoparks in China. The establishment of geoparks effectively protect precious and non-renewable geoheritage resources, as well as other natural, ecological and cultural landscape resources in an area. In addition, the construction of a nature reserve system with national parks as the main body has become one of the key tasks of China’s ecological civilization construction [97]. These measures have engendered a vital impact on the development of national and local tourism and the protection of the ecological environment. Huanggang Dabieshan UGGp should not only respond to national policy, but also explore a way suitable for its own development.

Firstly, balancing the conflict between human activities and ecosystem protection is the key to achieving the natural and socioeconomic sustainability of geoparks [76]. On the one hand, based on the existing overall plan, the administration should strictly implement the regulation of “no development in core protection areas and appropriate construction of tourism supporting facilities in non-core areas”, in order to abate negative effects on the ecological environment caused by human activities. On the other hand, the government needs to fully consider the coordination of local tourism policies and environmental protection policies, and gradually eliminate the weaknesses of tourism development and management. The government should also innovate environmental governance mechanisms, strengthen the construction of environmental treatment infrastructures, reduce the emission of various pollutants, and establish an early warning system for tourism ecological security.

Secondly, tourism enterprises need to be supported, and the investment of environmental protection funds should be increased. An ecological compensation mechanism should be set up with the support of regional public environmental protection finance.

Thirdly, promoting sustainable tourism, creating jobs and advertising local culture and products are some of the goals of UGGp [98]. Encouraging the participation of local communities in geotourism activities is instrumental in creating new employment opportunities and generating economic income for people living in rural areas [99]. When the geopark obtains economic benefits, it is bound to better increase the protection and economic strength, and thus local residents will pay more attention to protection because of the benefit from development.

Finally, it is essential to strengthen science popularization for the local public. Geoheritages, other natural resources, and cultural heritages are inheritances. Education in the form of teaching and entertainment can enhance the residents’ awareness of geoheritages and ecological environmental protection, which is conducive to enabling them to spontaneously maintain and improve the ecological environment of Huanggang Dabieshan UGGp.

### 4.6. Limitations

Some limitations need to be explained here. Due to the limitation of the data, this study only analyzed the data from 2000, 2005, 2010, 2015 and 2018. It is difficult to obtain the township-level data corresponding to some indicators, so that other data with similar meanings are used instead, which may cause deviation in the results. The variety and typicality of the data need to be further improved. The assessment of the greatest threats to the values of geopark may also affect TESI to some extent. It might be helpful to take the assessment into consideration. In addition, only one geopark is selected for evaluation, and the research scale is small. Consequently, further studies are essential to compare and analyze tourism ecological security in different geoparks in order to explore the overall spatio-temporal pattern of tourism ecological security of geoparks in China. It will be effective to master the driving mechanism of tourism ecological security of geoparks, and provide a theoretical reference for management strategies and sustainable development of geoparks.

## 5. Conclusions

On the basis of the DPSIR model, this paper constructs the tourism ecological security evaluation indicator system for the geopark. In this paper, the entropy weight method, comprehensive index method, spatial autocorrelation and obstacle degree model are used to examine the tourism ecological security of Huanggang Dabieshan UGGp. It analyzes the spatial and temporal evolution pattern and the influencing factors of TESI in the study area from 2000 to 2018. The conclusions are as follows:

1. The TESI of Huanggang Dabieshan UGGp shows a steady growth trend. During 2000 to 2005, the TESI was generally low in all the townships. In 2010, the TESI entered a critical safety level, and by 2018, the TESI had reached a relatively safe level. Especially, the TESI is higher in the townships where tourism resources are concentrated, tourism infrastructure is perfect, and tourism economy is highly developed.
2. The results of spatial autocorrelation analysis illustrated that the spatial agglomeration degree in Huanggang Dabieshan UGGp had shown a trend of first slowing down and then strengthening from 2000 to 2018. It indicated significant global and local spatial aggregation characteristics, and the overall pattern tended to be stable. The townships with different TESI levels represented obvious zone effects in spatial distribution, which showed the law of spatial decline. The TESI of townships where the main tourist attractions were located were at a high level, and the TESI of surrounding townships were at a low level.
3. Through obstacle analysis, it can be seen that the main obstacle factors included per capita tourism income, proportion of comprehensive tourism revenue in GDP, per capita net income of rural residents, proportion of tertiary industry in GDP, coverage of nature reserves, planning integrity of geopark, informatization of geopark, growth rate of tourists, comprehensive utilization rate of solid waste, etc. National policies, environmental governance, tourism load level, tourism development level, and geopark management have different impacts on the tourism ecological security in different periods.

## Figures and Tables

**Table 1:** Evaluation indicator system for tourism ecological security.

First-Level Indicator	Second-Level Indicator	Number	Unit	Attribute	Weight
Driving	Growth rate of natural population	X_1_	‰	−	0.0256
	Per capita GDP	X_2_	dollar	−	0.0211
	Urbanization rate	X_3_	%	−	0.0034
	Growth rate of tourists	X_4_	%	−	0.0237
	Growth rate of comprehensive tourism revenue	X_5_	%	−	0.0108
Pressure	Population density	X_6_	per/km^2^	−	0.0048
	Density of tourism economy	X_7_	ten thousand yuan/km^2^	−	0.0098
	Annual average concentration of SO_2_	X_8_	μg/m_3_	−	0.0312
	Annual average concentration of NO_2_	X_9_	μg/m_3_	−	0.0126
	Annual average concentration of inhalable particulate matter (PM_10_)	X_10_	μg/m_3_	−	0.0316
State	Regional development index	X_11_	%	−	0.0144
	Compliance rate of air quality	X_12_	%	+	0.0301
	NDVI	X_13_		+	0.0141
Impact	Per capita tourism income	X_14_	dollar	+	0.1038
	Proportion of comprehensive tourism revenue in GDP	X_15_	%	+	0.0654
	Per capita net income of rural residents	X_16_	dollar	+	0.0639
	Proportion of tertiary industry in GDP	X_17_	%	+	0.0337
Response	Domestic waste treatment rate	X_18_	%	+	0.0654
	Sewage treatment rate	X_19_	%	+	0.0316
	Comprehensive utilization rate of solid waste	X_20_	%	+	0.0418
	Coverage of nature reserves	X_21_	%	+	0.1684
	Proportion of education expenditure in GDP	X_22_	%	+	0.0234
	Planning integrity of geopark	X_23_	%	+	0.0541
	Interpretive coverage of geopark	X_24_	%	+	0.0599
	Informatization of geopark	X_25_	%	+	0.0555

Note: “+” indicates positive indicator and “−“ indicates negative indicator.

**Table 2:** Classification of TESI level.

Level	I	II	III	IV	V
**Category**	Unsafe	Less unsafe	Critical safe	Relatively safe	Safe
**Range**	(0, 0.2]	(0.2, 0.4]	(0.4, 0.6]	(0.6, 0.8]	(0.8, 1]

**Table 3:** TESI of Huanggang Dabieshan UGGp from 2000 to 2018.

Year	2000	2005	2010	2015	2018
**TESI**	0.176	0.227	0.440	0.551	0.657
**Level**	I	II	III	III	IV

**Table 4:** Ranking of Obstacle factors of TESI.

Year	Item	Ranking							
		1	2	3	4	5	6	7	8
2000	Obstacle factor	X_21_	X_14_	X_15_	X_18_	X_16_	X_24_	X_25_	X_23_
	Degree of obstruction (%)	20.563	12.673	7.987	7.987	7.802	7.316	6.782	6.602
2005	Obstacle factor	X_21_	X_14_	X_18_	X_15_	X_16_	X_24_	X_25_	X_23_
	Degree of obstruction (%)	21.944	13.511	8.591	8.046	7.920	7.682	6.734	6.596
2010	Obstacle factor	X_14_	X_16_	X_15_	X_20_	X_17_	X_25_	X_4_	X_23_
	Degree of obstruction (%)	23.379	11.691	10.808	10.015	8.093	5.977	4.054	3.853
2015	Obstacle factor	X_14_	X_8_	X_4_	X_15_	X_1_	X_20_	X_16_	X_17_
	Degree of obstruction (%)	18.334	10.816	8.198	8.017	7.363	7.306	6.118	6.052
2018	Obstacle factor	X_20_	X_1_	X_2_	X_11_	X_22_	X_7_	X_8_	X_12_
	Degree of obstruction (%)	18.445	17.702	14.550	9.928	7.779	6.768	5.756	5.486

## Data Availability

Not applicable.
